# Differentiation of Capripox Viruses by Nanopore Sequencing

**DOI:** 10.3390/vaccines9040351

**Published:** 2021-04-06

**Authors:** Kamal H. Eltom, Anna Christina Althoff, Sören Hansen, Susanne Böhlken-Fascher, Ausama Yousif, Hussein A. El-Sheikh, Ahmed A. ElWakeel, Mahmoud A. Elgamal, Hadeer M. Mossa, Emad A. Aboul-Soud, Janika Wolff, Christian Korthase, Bernd Hoffmann, Nabawia M. Adam, Sanaa A. Abdelaziz, Mohamed A. Shalaby, Ahmed Abd El Wahed

**Affiliations:** 1 Unit of Animal Health and Safety of Animal Products, Institute for Studies and Promotion of Animal Exports, University of Khartoum, Shambat 13314, Khartoum North, Sudan; kamal@uofk.edu; 2 Division of Microbiology and Animal Hygiene, University of Goettingen, D-37077 Goettingen, Germany; Anna.Althoff@wlv.de (A.C.A.); hansensoer@gmail.com (S.H.); sboelkenfascher@uni-goettingen.de (S.B.-F.); 3 Department of Virology, Faculty of Veterinary Medicine, Cairo University, Giza 12211, Egypt; ausama_yousif@cu.edu.eg (A.Y.); mhmoudelgaml@yahoo.com (M.A.E.); mshalaby43@gmail.com (M.A.S.); 4 Department of Infectious Diseases, Faculty of Veterinary Medicine, Zagazig University, Zagazig 44519, Egypt; husseinelsheikhvet@gmail.com; 5 Department of Epidemiology, Veterinary Medicine Directorate, General Organization of Veterinary Services, Benha 13511, Egypt; ahmedabdelhaleem946@yahoo.com; 6 Department of Poxvirus Vaccines, Veterinary Serum and Vaccine Research Institute, Abbasia, Cairo 11517, Egypt; hadeervsvri@gmail.com (H.M.M.); emadaboelsouad@gmail.com (E.A.A.-S.); 7 Institute of Diagnostic Virology, Friedrich-Loeffler-Institut, Federal Research Institute for Animal Health, Südufer 10, 17493 Greifswald-Insel Riems, Germany; janika.wolff@fli.de (J.W.); christian.korthase@fli.de (C.K.); bernd.hoffmann@fli.de (B.H.); 8 Department of Microbiology, Faculty of Veterinary Medicine, University of Khartoum, Shambat 13314, Khartoum North, Sudan; nabawiavet02011@gmail.com (N.M.A.); sabdelaziz262@gmail.com (S.A.A.); 9 Institute of Animal Hygiene and Veterinary Public Health, Faculty of Veterinary Medicine, University of Leipzig, An den Tierkliniken 43, D-04103 Leipzig, Germany

**Keywords:** lumpy skin disease, capripox virus, nanopore sequencing

## Abstract

The genus capripoxvirus (CaPV), family *Poxviridae*, includes three virus species: goatpox virus (GPV), sheeppox virus (SPV) and lumpy skin disease virus (LSDV). CaPV causes disease outbreaks with consequent economic losses in Africa and the Middle East. LSDV has recently spread to Southeast Europe. As CaPVs share 96–97% genetic similarity along the length of the entire genome and are difficult to distinguish using serological assays, simple, reliable and fast methods for diagnosis and species differentiation are crucial in cases of disease outbreak. The present study aimed to develop a field-applicable CaPV differentiation method. Nanopore technology was used for whole genome sequencing. A local database of complete CaPV genomes and partial sequences of three genes (RPO30, P32 and GPCR) was established for offline Basic Local Alignment Search Tool (BLAST). Specificities of 98.04% in whole genome and 97.86% in RPO30 gene runs were obtained among the three virus species, while other databases were less specific. The total run time was shortened to approximately 2 h. Functionality of the developed procedure was proved by samples with high host background sequences. Reliable differentiation options for the quality and capacity of hardware, and sample quality of suspected cases, were derived from these findings. The whole workflow can be performed rapidly with a mobile suitcase laboratory and mini-computer, allowing application at the point-of-need with limited resource settings.

## 1. Introduction

The genus capripoxvirus (CaPV), family *Poxviridae*, is composed of the three highly contagious virus species: (1) lumpy skin disease virus (LSDV), affecting mainly cattle; (2) goatpox virus (GPV), affecting goats; and (3) sheeppox virus (SPV), affecting sheep [1]. CaPVs are endemic in the African continent, and LSDV has spread to Russia and Southeast Europe over the past few years [2,3]. However, recent reports from the Balkan region showed a decrease of 95% in outbreak numbers from 2016 to 2017 and a stop of spread within affected countries after vaccination [4]. In the Middle East, ring vaccinations have been established in 10 km radius zones around outbreak spots in Iraq and Egypt [3,5] to control the spread of the virus.

The mechanical transmission by vectors is likely to facilitate the rapid propagation of LSDV, while SPV and GPV are transmitted mainly by direct contact [2]. Biting and blood-sucking insects, such as stable flies, mosquitoes and many tick species, are reportedly involved in transmission by feeding on livestock and changing hosts frequently [6,7,8,9,10].

Although the severity of the diseases varies among affected animal breeds, considerable losses are mostly provoked by direct mortality, drop in milk production, emaciation, secondary infections, medication costs and trade restrictions [3,11,12]. An Ethiopian survey of 243 herds revealed a median economic loss of USD 1176 at herd level due to LSDV outbreaks, whereas a survey of 80 Indian farms affected by SPV and GPV indicated average annual income losses of 30–40% [12,13]. The tendency of spreading to virus-free countries and the high economic losses in affected herds have raised interest in the topic of disease control. Development and quality assurance of vaccines and epidemiological investigations are of high priority to decrease outbreak numbers and prevent further spread to disease-free countries; therefore, accurate and reliable differentiation methods are required [4,14,15].

The genetic similarity is around 96–97% within the genus CaPV and, therefore, the virus species cannot be distinguished using traditional molecular assays [16]. However, cross-infections and immunities have been observed despite usual host preferences [11,14,17]. For example, Isiolo and Kedong GPV strains are capable of infecting sheep, goats and cattle [18]. Nonetheless, reports of LSDV infecting sheep, or SPV and GPV infecting cattle, have not been recorded so far [19].

Many CaPV isolates turned out to be geographically adapted and divergent in host preference. Therefore, differentiation among field strains is gaining more importance in order to identify the exact cause of an outbreak, especially in virus-free countries [20]. Various conventional, real-time and restriction fragment length polymorphism- polymerase chain reaction (PCR) assays targeting RPO30, P32 and GPCR genes were developed [21,22,23,24,25]. Yet, PCR suffers from error rates, eventually causing lower specificity when working with rising sequence lengths. This in turn might lead to misdiagnosis, especially in the differentiation of highly similar CaPVs.

The most specific method for virus identification is sequencing. Nonetheless, published protocols still depend on the amplification of target genes *via* PCR, followed by amplicon sequencing [26,27]. Moreover, PCR requires sophisticated technology for accurate temperature control, devices of large size and heavy weight and, sometimes, complex and long protocols for library preparation. Thus, well-equipped central laboratories of high monetary value are still indispensable at the present point in time. Next-generation sequencing technologies like Ion Torrent and Illumina have been established for efficient whole genome analysis [28,29,30]. However, these methods rely on long procedures for sample preparation, preamplification using PCR, cumbersome workflow and massive investment in hard- and software systems, which limit the implementation of these technologies in low- and middle-income countries [31]. A promising method to evade these requirements is Nanopore sequencing, developed by Oxford Nanopore Technologies (Oxford, UK). For the procedure, a specially tailored, quick library preparation kit is available. The protocol involves the use of transposome complexes that are contained in reagents [32]. These ensure the cleavage of the DNA and attachment of barcoded transposase adapters. With the addition of sequencing adapters, better threadability of the DNA is ensured for entering the nanopore. This sequencing technology makes use of a protein nanopore embedded in a membrane which is set under voltage. As DNA passes through the pore, individual changes in ionic current are measured to identify the single nucleotides. This method can be performed by the MinION (Oxford Nanopore Technologies), a pocket-sized portable device of less than 100 g, with an integrated flow cell. Its handiness and light weight allow sequencing at the point of need, providing direct translation of current measurements to nucleotide sequences *via* USB to a laptop. In this way, sequences of long read lengths can be generated in real-time and analysed immediately [33,34]. The procedure can be implemented in a mobile suitcase laboratory that contains all tools for sample preparation. With electricity supplied by a solar panel and/or battery, there is no necessity for any further infrastructure [35].

In the present study, we established a Nanopore sequencing method applicable for differentiating CaPVs in the field. A rapid barcoding protocol was carried out for library preparation, along with offline BLAST for analysis.

## 2. Materials and Methods

### 2.1. Viral DNA

DNA samples in cell culture of LSDV Neethling vaccine strain V100, SPPV strain V104 and GTPV strain V103 were provided by the Friedrich-Loeffler-Institut, Greifswald-Insel Riems, Germany. Full details about the used strains has been published previously [36,37].

### 2.2. Sample Preparation and Extraction

DNA was extracted from viral cell cultures using the QIAamp DNA Blood Mini Kit (QIAGEN, Hilden, Germany) as indicated in the manufacturer’s instructions. The DNA quantity was measured by Nanodrop ND-1000 spectrometer (Thermo Scientific, Waltham, MA, USA).

### 2.3. Library Preparation and Sequencing

For library preparation, the SQK-RBK004 kit and protocol for rapid barcoding (Oxford Nanopore Technologies, Oxford, UK) were used as recommended by the manufacturer. Briefly, barcoding was performed by mixing 7.5 µL containing a minimum of 400 ng template DNA together with 2.5 µL Fragmentation Mix. In this step, cleavage of the DNA template and attachment of the barcoded transposase adapters to the DNA is accomplished by transposome complexes stored in the Fragmentation Mix [26]. Samples were barcoded in the following order: barcodes 1 and 2, LSDV; 3 and 4, GPV; 5 and 6, SPV. The samples were pooled afterwards and mixed at equal volumes after applying AMPure XP beads (Beckman Coulter, Brea, CA, USA) for concentration. Then, 1 µL of Rapid Adapter was added to the sample to enhance threading of the DNA by attachment of sequencing adapters. The Flow-Cell was primed by the Flush Tether and Flush Buffer containing priming mix. Finally, the library was prepared by mixing 34 µL Sequencing Buffer, 25.5 µL Loading Beads, 4.5 µL nuclease-free water and 11 µL of DNA library before being loaded to the MinION Flow Cell 9.4, which was fitted on the MinION device (Oxford Nanopore Technologies, Oxford, UK), which was, in turn, connected to a laptop. The sequencing run was initiated using MinKNOW software (Oxford Nanopore Technologies) in the laptop. Sequence data produced from 5 min up to 12 h were saved as FAST5 files on the laptop; processing to FASTQ format and separation into barcodes were accomplished by the MinKNOW.

### 2.4. Offline Database and Data Processing

The software GENEIOUS 9.1.6 (Biomatter Ltd., Auckland, New Zealand) was used for establishing the local database and further analysis using offline BLAST. Nucleotide sequences of CaPVs were downloaded from GenBank of the National Centre for Biotechnology Information (NCBI) of the United States (https://www.ncbi.nlm.nih.gov/, accessed on 24 February 2021) and stored in libraries for offline use following a filtration by deleting sequences lower than 400 bp (full list of sequences is available online: https://doi.org/10.5281/zenodo.4559911, accessed on 24 February 2021).

The first database included various whole genome sequences of the three CaPVs. Out of these, a complete LSDV genome sequence was then sectioned into thirty, fifteen and ten regions of lengths 5, 10 and 15 kbp, respectively, which were extracted and used as additional databases. Furthermore, numerous virus-specific sequences of the genes RPO30, P32 and GPCR, which had been used for CaPVs differentiation in former studies [23,25], were retrieved from the NCBI database. A multiple alignment was run for the sequences of each gene. Subsequently, using the single-nucleotide polymorphism (SNP) detection tool, the sequences were examined in depth to identify regions with high occurrence of SNPs. The longest possible sequence of the identified region was extracted from suitable gene sequences of each virus. Thus, three virus specific extractions of each gene were used to set up the databases.

Offline BLAST of all databases was conducted for each barcode by the application of the MEGABLAST algorithm with an E-value of 10^−100^ and word size of 28. Results were displayed as query-centric alignment with a maximum of one hit per read. Specificities for the offline databases were concluded by the number of hits that were species-wise aligned correctly according to the viral origin of the barcoded reads, divided by the overall number of hits in the BLAST.

Online investigation was carried out using the What’s In My Pot (WIMP) tool of the EPI2ME desktop application of Oxford Nanopore Technologies. Average hits of each barcode were calculated by dividing the number of correctly identified sequences by the total number of identifications including all CaPV species.

The total sequence run was 12 h. In order to determine the shortest sequencing time needed to collect enough data to differentiate between CaPVs, offline BLAST on sequence files starting 5 min were analyzed.

### 2.5. Influence of Background

Pooled samples from endemic regions in Egypt and Sudan were used to test the influence of the background on the test performance: Egypt #1, pooled scabs from LSDV infected cattle; Egypt #2, pooled LSDV cultivated lab strain with cellular background; Egypt #3, a mixture of LSDV and SPV field strains; Sudan #1 and #2, suspected LSDV infection. All samples except Sudan #1 and #2 were confirmed to contain CaPV using molecular assay as described previously [38]. The threshold time is around 6 min in recombinase polymerase amplification assay.

## 3. Results

**Figure 1:**
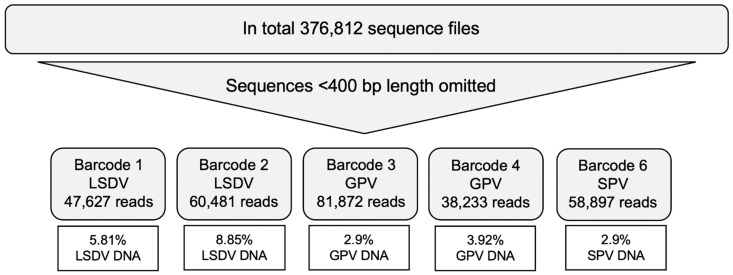
Filtration, classification and viral DNA amount of barcoded sequence files.

By sequencing of LSDV, GPV, SPV in duplicates, a total number of 376,812 sequence files were processed to FASTQ format and further analyzed in GENEIOUS and EPI2Me. After filtration (omitting sequences <400 bases), 287,110 reads were categorized into barcodes. Application of “map to reference” in GENEIOUS identified between 2.9% and 8.85% of the reads as CaPV sequences, depending on the barcoded sample (Figure 1). Barcode #5 was excluded as the number of reads were very low, mostly because of failure of binding of the assigned barcode during library preparation (full sequences are available online: https://doi.org/10.5281/zenodo.4559911, accessed on 24 February 2021).

**Figure 2:**
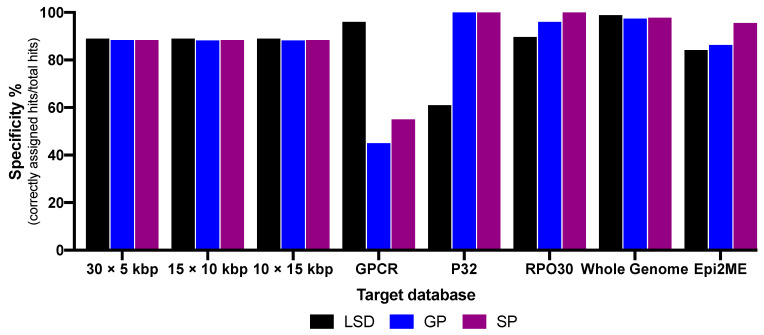
Average specificities of various offline Basic Local Alignment Search Tool (BLAST) and online EPI2ME databases.

Offline BLAST specificities of 5, 10 and 15 kbp genome regions, whole genome, RPO30, P32 and GPCR genes databases were calculated by dividing the number of hits that correctly aligned by the total number of hits (results are summarized in Figure 2). Whole genome database showed the best average specificity of 98.04%. Investigations utilizing the 15, 10 and 5 kbp genome regions revealed average pairwise identities ranging from 88.26% to 89.04% for the three viruses. In the gene databases, average specificities were 97.86%, 84.34% and 65.56% for RPO30, P32 and GPCR genes, respectively, while the online tool, WIMP (EPI2ME), showed an average of 84.48% of the reads mapped correctly to their respective virus species.

While the overall workflow had been initially conducted within 22 h (Table 1), it was functional when the sequencing time was reduced from 12 h to 25 min; the resulting total workflow became 2 h and 5 min (Table 2).

Pooled samples from endemic regions were used for validating the influence of the background on the nanopore sequencing and database performances (Table 3). Databases of whole genome and RPO30 gene as well as WIMP were applied for data analysis as they have shown the best specificity (Figure 2). The runs of the whole genome database identified Egypt #1 and #2 as LSDV, whereas Egypt #3 showed hits of LSDV and SPV. RPO30 BLAST did not show results for Egypt #1 and #2, while Egypt #3 revealed one SPV hit. WIMP mapped all Egypt samples as LSDV, with increasing amounts of Egypt #3 reads mapped as SPV. The software did not identify reads of the samples from Sudan as CaPV. Both offline BLAST based on the whole genome and the WIMP were the most accurate databases in the presence of host background.

## 4. Discussion

We were able to develop a highly specific differentiation method for the species of CaPV genus using Nanopore sequencing. The sequencing run was performed using a portable MinION device, while further differentiation was facilitated by offline local BLAST. Additionally, the WIMP online tool was tested.

The data analysis protocol is simple: the collected reads can be transferred to GENEIOUS software, which enables a simple handling in further data processing. All libraries needed for offline BLAST can be created by accessing the nucleotide database in the GenBank. The procedure can be prepared in advance by downloading CaPV sequences to GENEIOUS and creating databases of whole genome. In this way, point-of-need virus differentiation is enabled.

**Figure 3:**
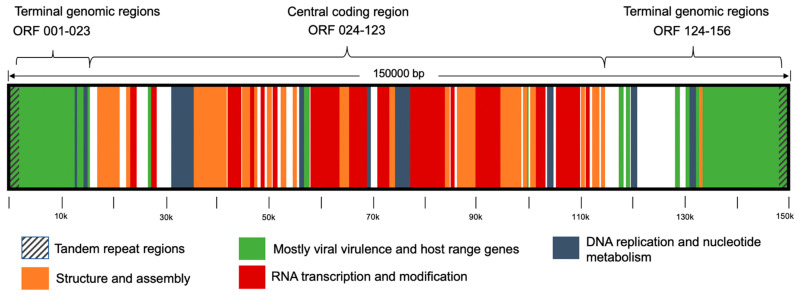
Capripox virus genome organisation, modified from Tulman et al., 2001.

CaPV genome is comprised of two noncoding identical inverted regions of tandem sequences (inverted terminal repeats) on the leftmost and rightmost sides bound to the central coding region. As mapped in Figure 3, the central genomic region contains mostly genes that encode replicative mechanisms and virion morphogenesis components. This region is enclosed by terminal genomic regions coding virulence and host range genes, for the most part [15,16]. Local BLAST was applied using whole genome sequences. Notably, all whole genome runs showed high specificities with an average of 98.04% of the sequences aligned correctly regarding the reads’ virus species. In the perspective of former studies that investigated genes in online sequence analysis [26,27], the use of whole genome sequences for offline analysis and differentiation is a novelty. This approach is eminently precise by aligning the 150 kbp long CaPV genome, yet it is adaptable to the variability of different isolates.

In order to detect genome regions that distinguish the virus species, multiple libraries of 15, 10 and 5 kbp long sequences taken from LSDV genome were established. For comparison, pairwise identities of the BLAST were examined. No significant divergences between the pairwise identities of LSDV and those of GPV and SPV were found. Therefore, none of the regions showed evidence of further genetic difference suitable for virus differentiation.

**Figure 4:**
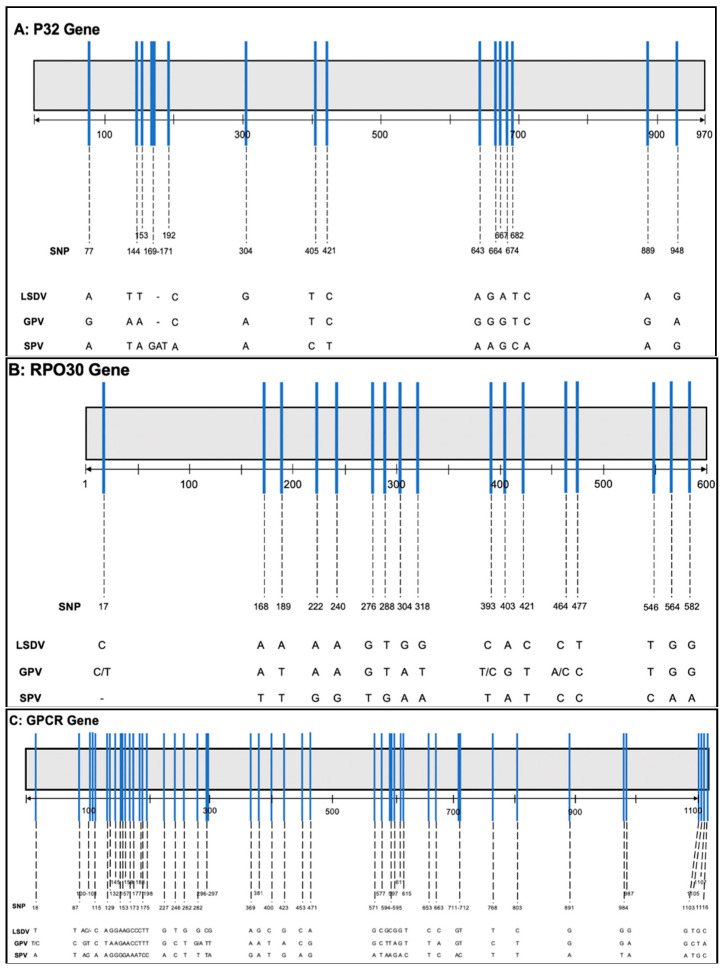
Single-nucleotide polymorphism (SNP) mapped to (**A**) P32 gene, (**B**) RPO30 gene and (**C**) GPCR gene. The single nucleotides of each position are shown below for LSDV, GPV and SPV.

For smaller database sizes, RPO30 gene proved high universal effectiveness with an average specificity of 97.86%. However, P32 and GPCR genes were not consistent in functionality. While P32 showed outstanding specificities of 100% in GPV and SPV, it had divergent results of 60.8% in LSDV. In contrast, GPCR gene was highly specific for LSDV, but of low specificity for GPV (39.7%) and SPV (55.8%). Positively correlated results of GPV and SPV in contrast to those of LSDV may most likely be affected by the genetic difference of the hosts, with adaption of GPV and SPV to animals of the caprine species and LSDV to bovines. These observations coincide with previous studies that found some genes in LSDV were disrupted in both GPV and SPV [16]. However, the difference in suitability might also be reasoned in gene function and structure (Figure 4A–C), as average specificity depends on gene size and number of SNPs. Thus, due to the low number of SNPs in RPO30 gene, divergences had a greater effect on the distinguishability of the viruses, whereas higher numbers of SNPs on the other genes caused a higher probability of being cancelled out by each other. Consequently, this resulted in more incorrect alignments and confusion in the differentiation. Additional approaches of combining different gene sequences in one database led to immense drops in specificity (data are shown in Supplementary File #1).

When the WIMP tool included in the desktop application EPI2ME of Oxford Nanopore Technologies was further tested as an online alternative, an average of 84.48% of correctly mapped sequences was obtained. Nevertheless, all barcodes have been identified successfully, proving reliability of the service. Moreover, the application provides a user-friendly interface with a more rapid result report than local BLAST. Considering the practicability in point-of-need implementation, WIMP depends on a stable internet connection, which is not always available, especially in rural areas. On the contrary, offline BLAST offered virus differentiation without this requirement. For validation of the used methods, whole genome and RPO30 databases were applied in further local BLAST using LSDV positive samples from Egypt and samples of LSDV suspects from the Sudan. While no hits were reported from both Sudan samples in all BLAST runs, samples #1 and #2 from Egypt were identified as LSDV, and sample #3 as both LSDV and SPV by the whole genome run. RPO30 database reported no hits in Egypt #1 and #2, and again SPV as identification of Egypt #3. Subsequently, WIMP tool was applied, which did not show CaPV-related results in Sudan #1 and #2. In fact, the previous results of local BLAST of Egypt samples corresponded to those of the WIMP analysis, showing major mapping to LSDV in Egypt #1 and #2, followed by high amounts of LSDV and increased mapping numbers of SPV in Egypt #3.

The local BLAST proved to be suitable for differentiation. Based on the obtained results, the whole genome BLAST database offers highly specific results among all CaPV species and is recommended to be performed using outstanding hardware facilities. In case of limited access to whole genome data, gene libraries provide high specificities and smaller size of the database. Depending on the case, genes can be applied interchangeably: in suspicion of LSDV, a combination of RPO30 and GPCR is recommended. For suspicion of GPV and SPV, RPO30 and P32 should be applied.

Furthermore, the online application WIMP can be used for accelerated result report. Although it performed successfully in this study, validation by offline BLAST is still advisable. However, despite its fast result report, the tool is still dependent on the availability of internet access.

Advanced infrastructure is a required component in the current state of diagnostics. Previously published protocols for CaPV differentiation that involved sequencing had been performed by target gene amplification using PCR, followed by cumbersome sequencing protocols and analysis by online algorithms [26,27]. Areas affected by CaPV outbreaks, however, are mostly lacking well-equipped, costly laboratories and steady internet connection. We have overcome these needs through the combination of Nanopore sequencing and offline BLAST. The use of a suitcase laboratory allows on-site sample preparation; with a size of 56 × 45.5 × 26.5 cm, it stores all of the tools and equipment required for the protocol [35]. The suitcase is shock absorbent and waterproof, and can be supplied with electricity by a solar panel. In this way, it is safe to transport and implement the equipment in low resource settings. Compared to PCR-based techniques, the usability of a suitcase system is superior due to its handiness and lower costs [35]. With sequencing conducted by a MinION device connected to a laptop, and sequence analysis performed using an offline algorithm, all requirements for CaPV differentiation are portable and field applicable, providing fast results. Furthermore, the online application WIMP can provide clues to the existence of other pathogens that may be contributing to observed disease conditions in sampled animals. Therefore, our method has the potential for versatile use in humble conditions and consequently enables accelerated diagnosis at the outbreak site.

## 5. Conclusions

In conclusion, we have accomplished the development of a fast and highly specific method for differentiation of CaPVs. The method is easy to replicate in laboratories or in a mobile suitcase laboratory for direct application in the field. Nevertheless, other clinically relevant issues must be addressed in future studies. This includes, but is not limited to, identification of clinical cases due to vaccination [39], differentiation between virulent and attenuated vaccine strains [40] and detection of the recombination events between vaccine and wild-type LSDV strains [41].

## Figures and Tables

**Table 1:** Speed of the workflow.

Step	Protocol Time		Kit/Program
	Short	Long	
Extraction	1 h		QIAamp DNA Blood Mini Kit
Library Preparation	20 min		Rapid Barcoding Sequencing Kit
Sequencing	25 min	12 h	MinION Flow Cell 9.4
Data Processing	15 min	8 h	MinKNOW
Analysis	5 min	30 min	GENEIOUS
Total	2 h 5 min	22 h	

**Table 2:** Comparison between the specificities of short (~2 h) and long (22 h) sequencing protocols.

Barcode	1 (LSD)		2 (LSD)		3 (GP)		4 (GP)		6 (SP)	
Run time	Short (~2 h)	Long (22 h)	Short (~2 h)	Long (22 h)	Short (~2 h)	Long (22 h)	Short (~2 h)	Long (22 h)	Short (~2 h)	Long (22 h)
Total number of Reads	1854	47,627	2174	60,481	3104	81,872	2158	38,233	2202	58,897
Specificity in % Whole genome BLAST	98.68	98.8	98.8	98.8	96.69	96.7	97.79	98	97.59	97.7

**Table 3:** Results of validation runs using samples from Egypt and Sudan, presented as percentage of mappings in offline BLAST and WIMP. Total number of correctly assigned hits is around 1% of the total number of reads. Most of the reads are background of host genome. The percentages in the table represent the specificities of the BLAST.

Sample (Total Reads)	Egypt #1 (17,912)	Egypt #2 (10,652)	Egypt #3 (11,268)	Sudan #1 (30,529)	Sudan #2 (6396)
Whole genome	95.08% LSDV	90.67% LSDV	74.19% LSDV 17.2% SPV	No results	
RPO30	No results	No results	SPV (1 Hit)		
WIMP	95.88% LSDV	84.54% LSDV	77.58% LSDV 19.82% SPV		

## Data Availability

All data are presented in the study and the full sequences as well as BLAST database available online: https://doi.org/10.5281/zenodo.4559911 Accessed on 24 February 2021.

## Supplementary Materials

The following are available online at https://www.mdpi.com/article/10.3390/vaccines9040351/s1. Supplementary file #1 contains the tables of the offline BLAST specificities of combining various genes.
