# Supplementary material for: Differentiation of Capripox Viruses by Nanopore Sequencing
Source: Vaccines (Basel). 2021 Apr 6;9(4):351. doi: 10.3390/vaccines9040351 (PMC8067513; doi:10.3390/vaccines9040351)
Supplement: Supplementary file 1 [file vaccines-09-00351-s001.pdf]

S_Table 1: Results of the combined BLAST database of RPO30 and P32 gene sequences. The library was established of sequences that served for the previous single gene BLAST databases.

Barcode	Specificity %
1	78.87
2	82
3	95
4	100
6	100

S_Table 2: BLAST search results of a database assembled of the best working gene sequences per virus. The library is comprised of RPO30 and GPCR sequences of LSDV, RPO30 and P32 sequences of GPV, RPO30 and P32 sequences of SPV.

Barcode	Specificity %
1	81.1
2	77.58
3	17.51
4	41.1
6	40.8
